# Morphological Characterization of Flower Buds Development and Related Gene Expression Profiling at Bud Break Stage in Heterodichogamous *Cyclocarya paliurus* (Batal.) lljinskaja

**DOI:** 10.3390/genes10100818

**Published:** 2019-10-17

**Authors:** Xiaoling Chen, Xia Mao, Peng Huang, Shengzuo Fang

**Affiliations:** Co-Innovation Center for Sustainable Forestry in Southern China, College of Forestry, Nanjing Forestry University, Nanjing 210037, China

**Keywords:** *Cyclocarya paliurus*, heterodichogamy, RNA-seq, differentially expressed transcription factors

## Abstract

*Cyclocarya paliurus* (Batal.) Iljinskaja, a unique species growing in southern China, is a multi-function tree species with medicinal, healthcare, material, and ornamental values. So far, sexual reproduction is the main method for extensive cultivation of *C. paliurus* plantations, but this is limited by low seed plumpness resulted from the character of heterodichogamy. Phenological observations have revealed the asynchronism of flower development in this species. However, its molecular mechanism remains largely unknown. To reveal molecular mechanism of heterodichogamy in *C. paliurus*, transcriptome of female (F) and male (M) buds from two mating types (protandry, PA; protogyny, PG) at bud break stage were sequenced using Illumina Hiseq 4000 platform. The expression patterns of both 32 genes related to flowering and 58 differentially expressed transcription factors (DETFs) selected from 6 families were divided four groups (PG-F, PG-M, PA-F, and PA-M) into two categories: first flowers (PG-F and PA-M) and later flowers (PA-F and PG-M). The results indicated that genes related to plant hormones (IAA, ABA, and GA) synthesis and response, glucose metabolism, and transcription factors (especially in MIKC family) played significant roles in regulating asynchronism of male and female flowers in the same mating type. The expression of DETFs showed two patterns. One contained DETFs up-regulated in first flowers in comparison to later flowers, and the other was the reverse. Nine genes related to flowering were selected for qRT-PCR to confirm the accuracy of RNA-seq, and generally, the RPKM values of these genes were consistent with the result of qRT-PCR. The results of this work could improve our understanding in asynchronism of floral development within one mating type in *C. paliurus* at transcriptional level, as well as lay a foundation for further study in heterodichogamous plants.

## 1. Introduction

Heterodichogamy, a transitional form in the evolution of plants from monoecism to dioecism, exists in hermaphrodite plants including two types of bisexual individuals, protandry (flowering male first) and protogyny (female first). When one type’s pollen is released, the stigma of the other is available; then they switch roles. It is one of the common strategies for plant to avoid inbreeding [1]. This kind of reproductive system is not common in flowering plants and has been found in only 21 genera from 13 families so far [2,3], such as *Cyclocarya*, *Juglans* in Juglandaceae, and *Acer* in Aceraceae.

*Cyclocarya paliurus*, known as Qing-Qian-Liu due to the resemblance of its fruit clusters to a string of old Chinese copper coins, is a sole species in this genus [4]. Because of its diverse functions, *C. paliurus* has attracted a lot of attention [5,6,7]. However, the natural forest resources are mainly distributed in the mountain areas that are hard to reach or some nature reserves [8]. In addition, as a typical heterodichogamous plant, *C. paliurus* was characterized by high setting percentage but low seed plumpness in nature and plantation forests [9,10], which brings big challenges for breeding. Phenological observation suggested that the flowering characters of heterodichogamy in *C. paliurus* were the main reason for low plump seed index [11,12]. Therefore, it is important to understand the mechanism of differentiation and development of flower in the process of breeding. 

The process of flowering is a transition from vegetative growth to reproductive growth under the interaction and coordination of internal and external factors, forming a complex network system to regulate flower formation [13]. Currently, the comprehensive and mature studies on the flowering network of *Arabidopsis thaliana* include six pathways mainly. The seasonal changes of day length and temperature are associated with flowering controlled by the photoperiod and vernalization pathways. And the pathway of the ambient temperature would respond to the daily growth temperature. In addition, the impacts of age, automatic and gibberellin pathways are more independent of the environmental stimulation [14]. Meanwhile, many floral integrators and floral meristem genes in such network were identified, such as *FT* (flowering locus T), *AGL* (agamous-like) family, *AP1* (apetala 1), and *LFY* (leafy) [15,16,17]. Although these studies mainly focused on model plants, more fragmentary information obtained from other species [18,19,20] reveals that both the developmental processes and the underlying regulatory networks are largely conserved [21]. They can pave the way for future studies in other unclear flowering plants, even in heterodichogamous plants. Nowadays, publications referring to flowering characteristics of heterodichogamy are limited to phenological observations [3,10,22,23], anatomical studies [11,12,24] and a few nutrients studies [25]. Overall, the detailed molecular mechanism or genetic elements on regulation of floral organ differentiation and development in *C. paliurus* remains elusive. With the identification of more and more bioactive compounds and physiological functions in *C. paliurus* [26,27,28,29], the market demand for the leaves is increasing. Apart from asexual reproduction with little progress, sexual reproduction is clearly irreplaceable in extensive cultivation and breeding excellent varieties, which are limited by the character of heterodichogamy. Genes expressed differently in two mating types may play important roles in floral development, and large numbers of genes may involve floral organ initiation in *C. paliurus*. Male and female flower buds from two mating types at bud break stage were analyzed by RNA-seq to make up the poorly understand in molecular mechanism of heterodichogamy. The aims of this study were to explore what factors or genes regulate synchronism of heterosexual flowers in different mating types and asynchronism of heterosexual flowers in the same mating type. The findings from this study could provide not only approach for further studies on the molecular mechanism of flower development in *C. paliurus* and other similar plants but also a theoretical basis for developing *C. paliurus* plantations.

## 2. Materials and Methods

### 2.1. Plant for Sample Collection

Samples were collected from a provenance trial of 9-year-old *C. paliurus* located in Chuzhou, Anhui Province, China (32°21′N, 118°58′E), which consists of more than 800 individuals. Eighteen individuals, including 9 protogyny (PG) and 9 protandry (PA), were randomly selected and tagged for sampling.

**Figure 1:**
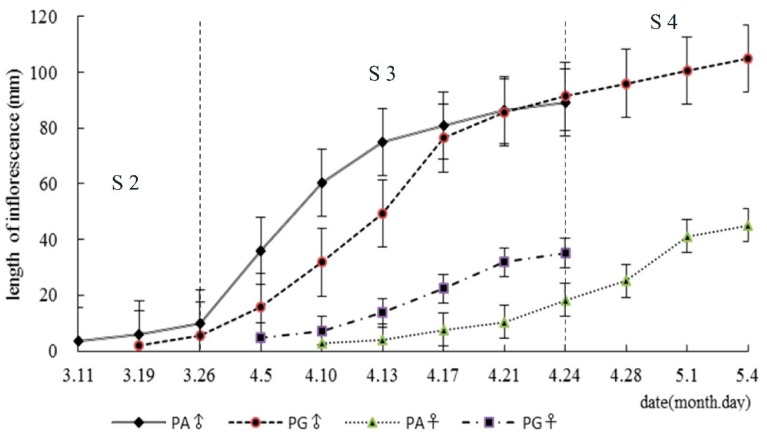
The elongation growth process of male and female inflorescence in two mating types of *Cyclocarya paliurus* [25]. PG = protogyny; PA = protandry.

Asynchronism in differentiation of male and female flower buds in two mating types is a typical characteristic in heterodichogamous plants. Based on the previous studies [10,25], flower bud differentiation and development were divided into five stages on the basis of the process of male differentiation in PA, which was the first sexual flower to start differentiation. Physiological differentiation stage (S0), which was invisible to the naked eye, initiated after fruit setting [30]. During S0, morphological differentiation of male buds in two types were rapidly completed, but female buds did not. Subsequently, floral buds entered a dormant period (S1) from September to early March of the following year. The next three stages were divided according to growth of inflorescences (shown in Figure 1): bud break stage (S2), inflorescence elongation stage (S3), and mature stage (S4).

In S2, morphological changes in male buds were observed first, indicating revival of plants. In addition, differential expression of genes involved floral bud development in S2 could elucidate the mechanism of heterodichogamy in *C. paliurus*. Thus, female floral buds (F) and male floral buds (M) were collected during S2 (March 15th, 2017) from two mating types and grouped into PG-F, PG-M, PA-F, and PA-M, respectively, with three replicates for each group. Each replicate was collected from three individuals. More than 15 male flower buds were collected for each replicate of PG-M and PA-M, while at least 45 female flower buds were sampled for PG-F and PA-F. All isolated floral buds were frozen in liquid nitrogen immediately and stored at -80 °C.

### 2.2. RNA Extraction, cDNA Synthesis, and Sequencing

E.Z.N.A Plant RNA Kit (OMEGA, American) was used to extract and purify total RNA of each sample. The extracted RNA was divided into two parts, one for sequencing and the other for later verification. The concentration and quality of RNA were checked by NanoPhotometer® (IMPLEN, Los Angeles, CA, USA) and Qubit®3.0 Flurometer (Life Technologies, Boston, CA, USA). 2100 RNA Nano 6000 Assay Kit (Agilent Technologies, Santa Clara, CA, USA) was used to detect the integrity of RNA. Beijing Annoroad Genome Co. Ltd. constructed the cDNA library, performed sequencing on an Illumina platform, and generated 150 bp paired-end reads. 

### 2.3. Data Filtering, De Novo Assembly, and Functional Annotation

Raw Data was processed with Perl script to clean data by removing the adaptor-polluted reads containing more than 5 adaptor-polluted bases, the low-quality reads with percentage of low Q-value (<20) base higher than 15%, and reads with number of N bases more than 5%. Both reads would be filtered out if any read of the paired-end reads is unqualified. Then, clean reads were de novo assembled by software Trinity (http://trinityrnaseq.sourceforge.net/, Version number: trinityrnaseq, release-20140717) into unigenes [31].

TransDecoder identified candidate coding regions within transcript sequence. To understand function of unigenes and ORFs, assembled unigenes were annotated by Nr, Nt, SwissProt, PFAM, KOG, GO, and KEGG database using NCBI Basic Local Alignment Search Tool (BLAST) with an E-value ≤ 10^−5^ [32]. 

### 2.4. Differentially Expressed Genes Between Female and Male Buds in Two Mating Types 

The expression levels of unigenes were calculated by RPKM (Reads per kilobase million mapped), which could eliminate the effect of sequencing depth and gene length on gene expression levels, so that the data could be compared between each other directly. The levels of unigenes’ expression at different floral buds were calculated using DESeq method described by Anders and Huber [33].

### 2.5. Quantitative Real-Time PCR Verification

Unigenes related to flower bud differentiation were selected for qRT-PCR analysis. RNA left for verification was turned to cDNA according to the protocol of PrimeScript™ II 1st Strand cDNA Synthesis Kit (Takara, Dalian, China). Specific primers were designed by Primer Premier 5.0 software to ensure the length of amplified products are between 120bp and200bp. All reactions were performed using SYBR Green Realtime PCR Master Mix Kit (Toyobo, Osaka, Japan) by StepOne Real-Time PCR System (Applied Biosystems, Foster City, CA, USA) according to the manufacturer’s instructions. The experimental reaction conditions including denaturation at 95 °C for 30s and 40 cycles of denaturation at 94 °C for 10 s, annealing at 55 °C for 30 s, and extension at 72 °C for 40 s. Relative gene expression was calculated by the 2^−ΔΔCt^ method with 18S ribosomal RNA as an internal control gene [34].

## 3. Results

### 3.1. Morphological Characteristics of Floral Bud Development

**Figure 2:**
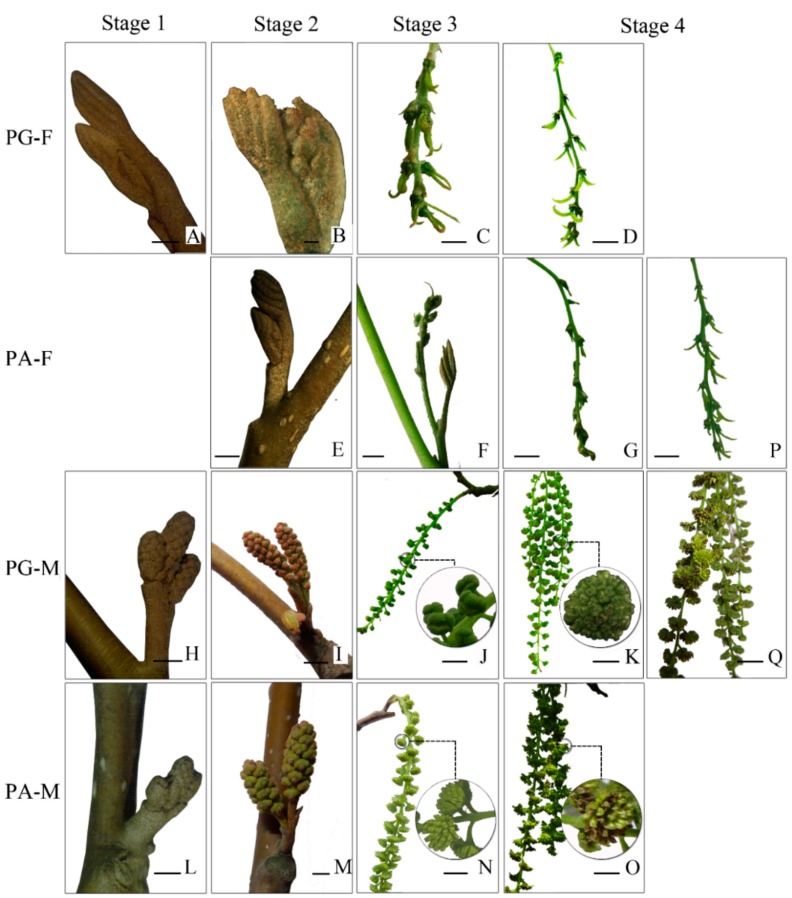
Morphological changes of flower development in *C. paliurus.* The bar is 0.2 cm (**B**); 0.5 cm (**A**, **E**, **H**, **L**); 1 cm (**B**, **J**, **M**);2 cm (**C**, **F**, **G**, **I**, **J**, **M**, **N**); 3 cm (**D**, **K**, **O**, **P**, **Q**). In S1, morphological characteristics of female buds was no difference with leaf buds (**A**, **E**); male buds clearly differentiated (**H**, **L**). In S2, all flower buds began to protrude, except PA-F (**B**, **I**, **M**). In S3, feathery stigma formed in PG-F (**C**), while it was not observed in PA-F (**F**); PA-M turned from turquoise to yellow-green with anther dehiscence (**N**); PG-M still remained turquoise (**J**). In S4, the feathery stigma of PG-F opened (**D**) and the mature pollen of PA-M was released from the dehiscent anther (**O**); the stigma of PA-F was not fully formed (**G**), and the anther of PG-M was not dehiscent (**K**). (**P**) Maturation of PA-F; (**Q**) maturation of PG-M.

*C. paliurus* is a typical heterodichogamous species, including two mating types (PG and PA). The process of floral bud differentiation and development was significantly different between two mating types. Obvious differences of floral bud development (S1–S4) in morphology of four groups were photographed (Figure 2). Generally, PG-F developed faster than PA-F, while PA-M developed faster than PG-M (Figure 1, Figure 2), in accord with the flowering rhythm of heterodichogamy. In both PG and PA, male buds had completed morphological differentiation but female buds had not, then they entered dormant stage (S1). During S2, inflorescences of PA-M and PG-M elongated at slightly different speeds (Figure 1); in the meantime, few inflorescences of PG-F protruded from surrounded leaf buds (Figure 2B), yet the female buds in PA were still invisible (Figure 2E). S3 was the elongating stage of inflorescences, at which elongation of PG-F and PA-M completed and rapid elongation of PG-M and PA-F occurred (Figure 1, Figure 2C,F,J,N). In the early period of S4, PG-F and PA-M matured and began to mate with each other, while, PG-M and PA-F were just finishing inflorescences elongation (Figure 1, Figure 2G,K), while the maturation of PG-M and PA-F was observed in the later period of S4 (Figure 2P,Q).

### 3.2. Sequencing, Assembly, and Annotation

**Figure 3:**
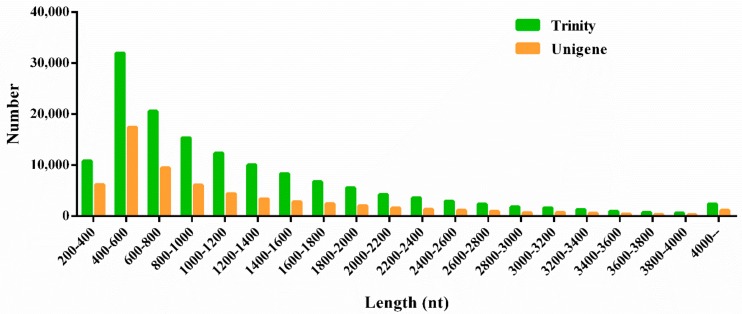
Pattern of length distribution of assembled trinities and unigenes.

High throughput RNA sequencing was obtained over 10 Gb clean data from each bud sample with Q30 all higher than 93.30%. All data are available from the NCBI Short Read Archive (SRA) with an accession number PRJNA553355. Assembly of the high-quality reads generated a total of 63,533 unigenes, with a N50 of 1586 bp and the average length of 1132.95 bp. The length distribution of unigenes and trinities was showed in Figure 3. Thirty-nine thousand, eight hundred and eighty-six unigenes were longer than 600 bp, and 9237 unigenes longer than 2000 bp. Unigenes between 400–600 bp were of the largest proportion.

A total of 46,009 unigenes were annotated by BLAST in 12 public databases (BLASTP, BLASTX, GO, COG, KO, NR, NT, PFAM, Prot, SignalP, TmHMM, and eggNOG), leaving approximately 27.6% unigenes without annotation. Unigenes up to 37375 (58.83%), 31330 (49.31%), and 26158 (41.17%) displayed high homology (1e < 10^−50^) to known sequences in NR, Prot, and BLASTX databases, respectively (Table 1).

Twenty-five thousand, one hundred and sixty-seven unigenes were divided into three categories (“metabolic process”, “cellular process”, and “catalytic activity”) and 66 functional groups based on GO classification (Table 1, SM1). Eleven thousand, three hundred and ninety-four unigenes annotated by COG classification were divided into 24 classes, and “general function prediction only” was defined the largest group with 1666 unigenes, followed serially by “Translation, ribosomal structure and biogenesis (1597)” and “Post-translational modification, protein turnover, chaperones (1233)”. However, groups of “Nuclear structure” only contained 2 unigenes (SM2).

### 3.3. Expression of Major Genes Related to Flower Differentiation

**Figure 4:**
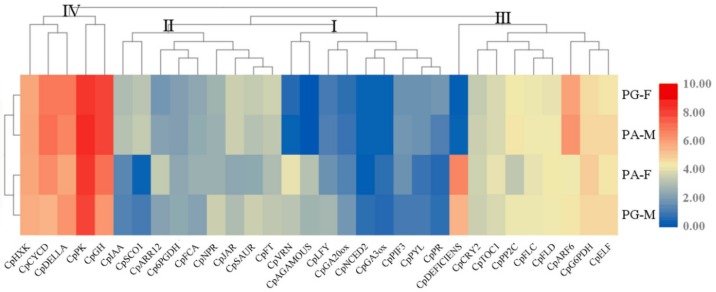
Heat map diagram of relative expression levels and classification based on the expression patterns of the related genes in flowering.

Thirty-two homologous genes in *C. paliurus* were detected to associate with flowering. According to RPKM, four groups were divided into two categories, first flowering type (PG-F, PA-M) and later flowering type (PG-M, PA-F) (Figure 4), by clustering analysis of 32 genes. Meanwhile, these homologous genes were divided into 4 expression levels marked with I, II, III, and IV from low to high. Level I meant the genes with low expression at S2, such as GA20-oxidase (*CpGA20ox*) and GA3-oxidase (*CpGA3ox*) related to synthesis of GA [35], *CpNCED* (9-cis-epoxycarotenoid dioxygenase) related to the synthesis of ABA [36], and transcriptional repressor VRN1 (*CpVRN1*) involve in vernalization [37]. Level II mainly contained genes associated with IAA (auxin-responsive protein IAA (*CpIAA*), SAUR family protein (*CpSAUR*)) [38] and other plant hormone (two-component response regulator ARR12 (*CpARR12*) [39], jasmonic acid-amino synthetase (*CpJAR*) [40], regulatory protein NPR1 (*CpNPR1*)) [41] signal transduction. Most genes associated with photoperiod pathway (cryptochrome circadian regulator 2 (*CpCRY2*) [40], early flowering (*CpELF*), timing of CAB expression 1 (*CpTOC1*)) [42] and vernalization pathway (flower lucos C (*CpFLC*) and flower lucos D (*CpFLD*)) [43] were assigned to level III. Level IV, the highest expression contained 2 genes (hexokinase (*CpHXK*) [44] and pyruvate kinase (*CpPK*)) [45] belonging to metabolism of glucose, 3 genes responding to GA (*CpDELLA*) [46], CK (cyclin D3 (*CpCYCD3*)) [47], and IAA (auxin responsive GH3 gene family (*CpGH3*)) [38], respectively. Particularly, the expression of *CpDEFICIENS* [48] in later flowering type was much higher than that in first flowering type. In addition, *CpARF6* (auxin response factor 6) [49] showed an obviously opposite expression pattern in two categories.

### 3.4. Identification of Differentially Expressed Genes

**Figure 5:**
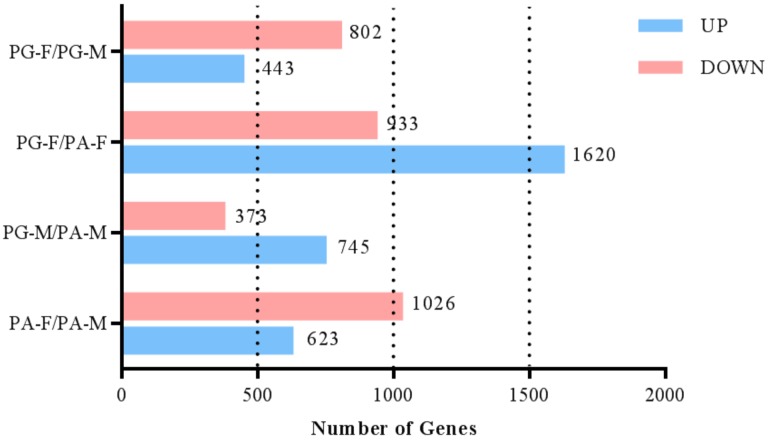
Numbers of differentially expressed genes in different floral buds of two mating types at bud break stage (S2).

Four pair-wise comparisons (PG-F vs PG-M, PG-F vs PA-F, PA-F vs PA-M, PG-M vs PA-M) were established among four groups (PG-F, PG-M, PA-F, PA-M) according to RPKM (Figure 5). DEGs were identified by |log2FoldChange| ≥1 and q ≤ 0.05. The greatest number of DEGs (2553) was detected in PG-F vs PA-F, with 1620 up-regulated and 933 down-regulated unigenes, and followed by PA-F vs PA-M (1649) with 1026 down-regulated unigenes and 623 up-regulated unigenes. In PG-F vs PG-M, 802 unigenes were down-regulated and 443 unigenes were up-regulated. The least number of DEGs was found in PG-M vs PA-M with 745 up-regulated and 373 down-regulated unigenes.

### 3.5. KEGG Pathway Annotation of Differentially Expressed Genes

**Figure 6:**
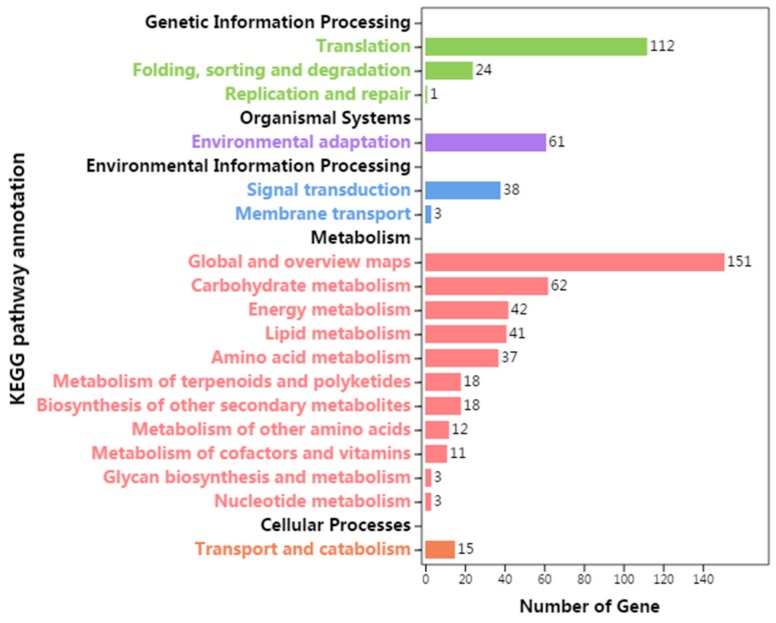
KEGG pathway annotation of differentially expressed genes (DEGs).

A total of 609 DEGs were annotated in 101 KEGG pathways, which were divided into 5 branches and 18 sub-branches (Figure 6). Four hundred and eight DEGs were associated with “metabolism”, and “global and overview maps (151)” was the greatest sub-branch in this item, followed by “carbohydrate metabolism (62)”. “Environmental adaptation” and “signal transduction” owned 61 and 38 DEGs, respectively. It is well known that “carbohydrate metabolism”, “signal transduction”, and “environmental adaptation” play significant roles in flower differentiation and development.

### 3.6. Major Differentially Expressed Transcription Factors

**Figure 7:**
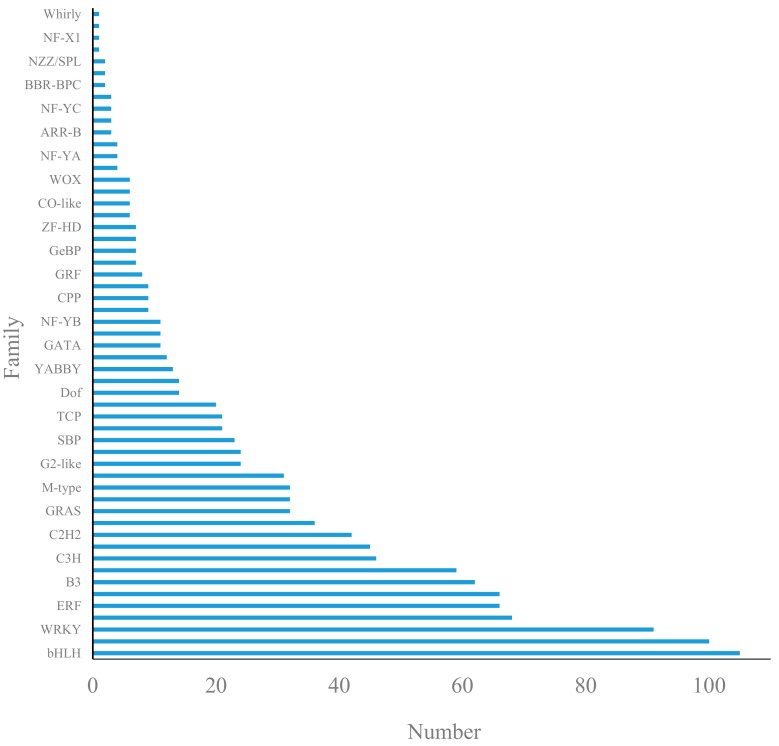
Differentially expressed transcription factors in floral buds of two mating types at bud break stage (S2).

One thousand two hundred and fifty-three TFs belonging to 55 families were identified through differentially expressed analysis among the four comparisons (Figure 7). The bHLH family occupied the largest portion, while up to 26.9% of TFs were involved in regulating flower development, including 6 families of MADS-box (MIKC), MYB, SBP, bZIP, and B3 (ARF and RAV plus) family.

**Figure 8:**
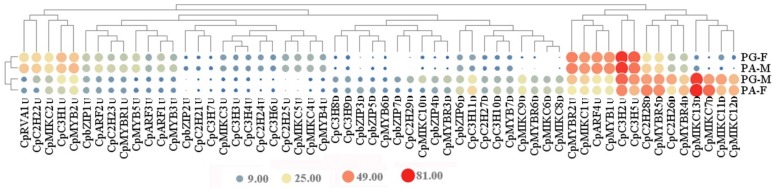
Heat map diagram of relative expression levels and classification based on the expression patterns of differentially-expressed transcription factors (DETFs) related with flowering. The name of each gene is suffixed with “U” or “D”. “U” represented up-regulated in the first flowers, “D” represented down-regulated; the circle size represented the level of expression, while red represented high expression, and blue represented low expression.

Fifty-eight DETFs related to flowering were detected by intersecting DETFs in the four pair-wise comparisons. DETFs with similar expression trends were clustered together, and the majority of genes in the same family were clustered together (Figure 8 and SM3). The expression of 58 DETFs displayed 2 patterns, one expressed with high level in first flowers (PG-F and PA-M) but with low level in later flower (PG-M and PA-F), and the other displayed an opposite pattern. DETFs from 6 TF families showed both patterns, with the exception that 2 sub-families (ARF and RAV) belonging to B3 family were of only one pattern (higher expression in first flower). Generally, the majority of DETFs showed slight differences at a low expression level, while, some genes from MIKC family showed very distinct differences, especially for *CpMIKC7D* and *CpMIKC13D*.

### 3.7. Validation of RNA-Seq Result by qRT-PCR

**Figure 9:**
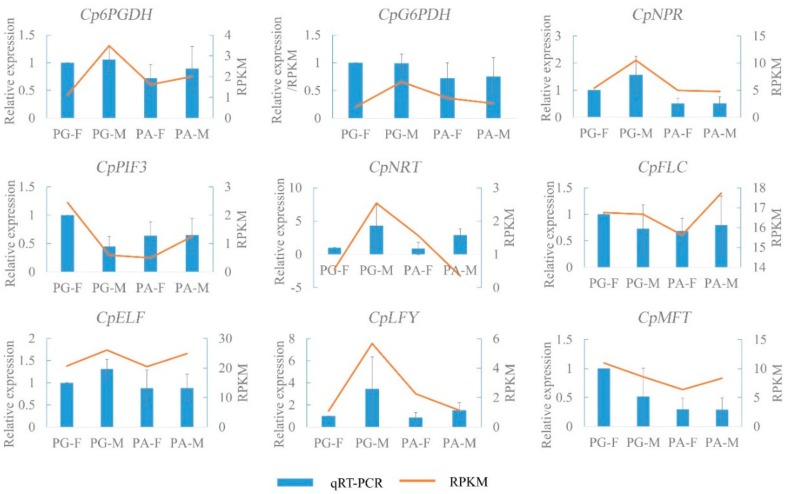
Comparison of expression levels of 9 genes obtained by qRT-PCR analysis and RNA-seq (RPKM (Reads per kilobase million mapped) values). The error bar is SD (standard deviation) for the three replicates.

To confirm the accuracy of RNA-seq results, 9 candidate genes involved in flowering process were selected for qRT-PCR analysis (Figure 9). Among them, 2 genes (6-phosphogluconate dehydrogenase (*Cp6PGDH*)-c34561_g4, glucose 6-phosphatedehydrogenase (*CpG6PDH*)-c41263_g2) were related to pathways of starch and sucrose metabolism [50,51], 2 genes (phytochrome interacting factors 3 (*CpPIF3*) [52]-c31958_g1, *CpNPR*-c34174_g1) involved plant hormone signal transduction, 1 gene (*CpFLC*-c40321_g2) was regulated by vernalization, and the other 3 genes (*CpELF* -c36049_g1, *CpLFY*-c25961_g3, mother of FT (*CpMFT*) [52]-c26300_g1) involved flowering process. 

The expression patterns of 9 genes in different samples obtained by qRT-PCR generally were consistent with RPKM values from RNA-seq (Figure 9), indicating the expression profiles of our transcriptome results were reliable.

## 4. Discussion

Multi-sex strategy is an important approach to plant evolution. There are two or more mating types in some plant groups. The differences of sex morphology and flowering phenology lead to non-random mating. Disassortative mating is often considered to be necessary in the evolution of sexual diversity [53], while heterodichogamy is an important type of disassortative mating. Angiosperm evolution of dioecy (separate male and female plants) from monoecism (presence of male and female in the same flower) resulted either through the successive differentiation of male and female flowers, or through the monoecious flowers showing dioecious type of mating system [54]. Usually, mating take place between the plants rather than within the plant. Therefore, heterodichogamy is considered to be a transitional form in the evolution of plants from monoecism to dioecism [55].

Although heterodichogamy plays an important role in plant evolution and cultivation of woody economic plants, more researches have only focused on descriptions of flowering phenology [3,10,22,23] and anatomical observations [11,12,24]. However, intrinsic mechanism controlling heterodichogamy are still unknown. As an important medicinal and typically heterodichogamous plant, *C. paliurus* has attracted more interests. Besides publications on functions and accumulations of various secondary metabolites [26,27,28,29], many researches on *C. paliurus* mentioned above, laid the foundation for further understanding the heterodichogamy on molecular level. Differences in both morphology and physiology (nutrients and hormones) were monitored throughout whole developmental process of male/female flowers in two mating types of *C. paliurus* [25]. Of five stages of flower differentiation and development, physiological differentiation stage (S0) and bud break stage (S2) are two key stages affecting further asynchrony. Compared to invisibility of buds at S0, transcriptome analysis of various sexual flower buds in two mating types of *C. paliurus* (PA-M, PG-F, PA-F, and PG-M) at S2 could explain the changes in visible morphology.

### 4.1. Expression Pattern of Major Genes in Different Pathways of Forming Flowers

According to Tair (https://www.arabidopsis.org/) and flowering network from other plants, 32 genes in *C. paliurus* directly or indirectly took part in different pathways of forming flowers, including glycometabolism, autonomous pathway, photoperiod pathway, and GA pathway (Figure 5). Genes in autonomous pathway (RNA-binding protein FCA (*CpFCA*)) [56] and photoperiod pathway (*CpCRY2*, *CpTOC1*) showed almost no difference in male and female flower buds, which manifested that these genes respond similarily at S2 (Figure 5). In the vernalization pathway, not *CpFLC* itself but the inhibitor of *CpFLC*–*CpVRN* highly expressed in later flowers (PG-M and PA-F), showing the degree of inhibiting *CpFLC* by vernalization was different in two categories. *CpDEFICIENS*, a transcription factor involving in genetic control of flower development that acts in conjunction with floral homeotic protein GLOBOSA (*GLO*), showed a much higher expression in later flowers, and *CpAGAMOUS* displayed a similar expression pattern. These genes could have contributed to the early flower differentiation, which can be verified by subsequent studies.

Glycometabolism is the basis of plant growth and development for providing energy and materials [57], especially in the process of flower bud development. Except for *Cp6PGDH*, other genes associated with glycometabolism showed high expression, especially *CpPK* as a key rate-limiting enzyme in the final step of glycolysis. *Nicotiana tabacum* with *PKc* deficient grew weaker under weak light, additionally, differentiation of flower bud and flowering in the mutant were significantly delayed compared to wild type [45]. Although with high expression, these genes did not show significant differential expression between the first flowers (PG-F and PA-M) and the later flowers. The expression patterns of these genes in flower buds also support the results of Huang et al. [25], who revealed that soluble sugar for flower development derived from surrounding branches rather than itself in *C. paliurus*. It could be inferred that these expression pattern in two mating types might be independent of activity of glycometabolism in flower buds themselves, but be related to the amount of soluble sugar transported by surrounding branches. And, the high expression of these genes in flower buds might be for the next developmental stage (inflorescence elongation stage, S3).

In addition, hormones play an equally important role in flower bud differentiation and development. Generally, the initiation and termination of dormancy are regulated by hormones, and environmental factors also affect dormancy by regulating hormone synthesis and transport. The pathway of plant hormone signal transduction was significantly enriched through KEGG enrichment analysis. Combined with expression of genes in such pathway, genes responding to IAA (*CpIAA*, *CpARF6*, *CpGH*) and JA (*CpJAR*) had higher expression in first flowers. It is consistent with previous results that the average content of IAA in first flowers (7.474 ng/gFW in PG-F and 5.824 ng/gFW in PA-M) was higher than that in later flowers (NC in both PG-M and PA-F) [58]. This suggests that IAA might help flower buds to break dormancy and promote inflorescence elongation. Genes responding to CK (*CpARR12*) and ABA (protein phosphatase 2C (*CpPP2C*) [59]) showed the opposite expression level (*CpARR12* with the highest level and *CpPP2C* with the lowest level) in PA-F (Figure 5), indicating that they could play the completely different roles in dormancy releasing of flower buds. However, the lowest content of ABA was found in PG-F (20.016 ng/gFW), compared with PG-M (134.478 ng/g FW), PA-F (52.893 ng/g FW), and PA-M (95.381 ng/g FW) [58]. Many researches have showed that ABA acted as a floral repressor, and the mechanism of inhibiting flowering involved interaction with other proteins (*DELLA*) or transcription factors (*FLC*) [60,61]. The inconsistency between responsive protein (*CpPP2C*) and content of ABA might be that *CpPP2C* is not the sole receptor of ABA, and more detailed studies are required to illustrate the relationship between ABA and flowering. 

One of major pathways controlling the time of flowering is GA pathway, and GA had important effects on breaking dormancy of flower buds [62,63,64]. For instance, Zhuang et al. [65] found that flower buds of Japanese apricot treated with GA_4_ released dormancy 30 days earlier. In *C. paliurus* at S2 stage, low expression level for genes related to GA synthesis (*CpGA3ox* and *CpGA20ox*) was found in all groups; whereas *CpDELLA*, one of gibberellin responses, showed high expression level in four groups, and relative lower in PA-F, which demonstrated that the synthesis of GA was mainly on the eve of buds breaking. Alternatively, GA synthesis might happen in other tissues then be transported to flower buds [66].

### 4.2. Expression Pattern of TFs Related to Flowering from DEGs

Heterodichogamous plants display the asynchronism of flower developmental process rather than in morphology under similar environmental conditions, suggesting that the regulatory regions of genes controlling the development of flowering should get more attention in further work. TFs could interact directly with chromatin factors and promote its recruitment to the related genes to determine appropriate timing of floral organ patterning [67,68]. And many TFs were proved to associate with flowering in some plants, including MIKC-type proteins, MYB, bZIP, Zinc-finger transcription factor, and so on [69,70,71,72,73].

TFs involving flowering were selected from DEGs in this study. According to the differences of expression patterns, DETFs were divided into two categories. One contains DETFs up-regulated in first flowers in comparison to later flowers, and the other is the reverse (Figure 8). Besides, the majority of DETFs with lower expression level showed slight differences between first flowers and later flowers. Many TFs belonging to same family showed similar expression pattern, especially those in B3 family (including ARF and RAV). The ARF family consists of auxin response factors, and they work by binding auxin and auxin responsive protein (Aux/IAA) responding to auxin signaling to regulate the early expression of auxin response genes [74]. All 4 TFs (*CpARF1*U-auxin-responsive protein IAA7, *CpARF2U*- caffeoyl-CoA acyltransferase-like, *CpARF3U*- auxin response factor 4-like, *CpARF4U*- auxin response factor 6-like) in ARF selected in our study showed higher expression in first flowers, in accord with their rapid developmental process. MYB and MIKC family showed irregular expression patterns in the first and later flowers, inferring they might have a wide range of functions. 

Even the same TF, distinct expression levels were monitored between first and later flowers. It is worth noting that several DETFs with the most differences are from MIKC family. Most of the genes in the "ABC" model of flower development in *A. thaliana* belong to the MIKC family [75]. MIKC-type proteins, belonging to MADS-domain transcription factors, play the crucial roles in controlling floral organogenesis and flowering time in plants [69]. MIKC-type consists of MIKC* and MIKC^C^. MIKC^C^ type proteins contain 13 clades, among which 12 clades were found in *A. thaliana*, such as *AG*, *AGL2*, and *AGL6* [76]. Of DETFs in *C. paliurus*, 13 DETFs (*CpMIKC1-13*) were annotated as *SVP2*, *AGL24*, *AGL42*, *AGL11*, *AGL42*, *AGAMOUS*, *DEFICIENS*, *AGL15*, *AGL9*, *AGL6*, *AP1*, *AGL2* and floral homeotic protein PMADS 2-like, respectively. The functions of many TFs in MIKC have been confirmed to be associated with flowering. For example, high expression of *AGL15* resulted in a decrease in the amount of active GA in transgenic *A. thaliana*, leading to late flowering [77]. This is consistent with our results that the expression of *AGL15* (*CpMIKC8D*) in later flower was higher than in early ones. *CpMIKC6D*, annotated as *AGAMOUS*, a key regulator of flower organ development in *A. thaliana*, determines the formation of reproductive organs and gender differentiation and fruiting [78]. 

Recent studies have demonstrated that MADS family also involved bud dormancy and germination, like *DAM* (dormancy-associated MADS-box), which is an important gene to study the relationship between MADS-box and bud dormancy. The expression of *DAM* increased before breaking dormancy, and decreased after breaking dormancy [79,80,81]. Furthermore, in *Cerasus avium*, the expression of *AGAMOUS* up-regulated along with increase of GA_3_ concentration [82], indicating that it could be the synergistic effect with gibberellin on breaking dormancy. The expression pattern of *CpAGAMOUS* in first flowers and later flowers seems to support above speculation (Figure 8). Floral homeotic protein PMADS 2-like (*CpMIKC13D*), *DEFICIENS* (*CpMIKC7D*), *AP1* (*CpMIKC11D*), and *AGL2* (*CpMIKC12D*) showed the most significant difference between the two categories, and all of them shared the same expression pattern with *CpAGAMOUS*, suggesting they could also play a role in regulating flower development through the gibberellin pathway. They are good clues for further study to reveal TFs functions on the molecular mechanism of heterodichogamy.

## Figures and Tables

**Table 1:** Summary of unigenes annotation.

Database	Count	Percentage (%)
BLASTP	22,563	35.51
BLASTX	26,158	41.17
GO	25,167	39.61
KO	11,027	17.36
NR	37,375	58.83
NT	21,777	34.28
PFAM	23,464	36.93
Prot	31,330	49.31
SignalP	2154	3.39
COG	11,394	17.93
TmHMM	6488	10.21
eggNOG	13,476	21.21
Total annotation	46,009	72.42
Total unigenes	63,533	100
